# ﻿Two new species of *Hyalella* (Amphipoda, Dogielinotidae) from the Humid Chaco ecoregion of Paraguay

**DOI:** 10.3897/zookeys.1191.113840

**Published:** 2024-02-12

**Authors:** Giovanni Mussini, Nicole D. Stepan, Gersey Vargas

**Affiliations:** 1 Department of Earth Sciences, Downing Street, University of Cambridge, CB2 3EQ, Cambridge, UK University of Cambridge Cambridge United Kingdom; 2 Colección Científica Para La Tierra (CCPLT), Fundación Para La Tierra, Centro IDEAL, 321, Mariscal José Félix Estigarribia, c/ Teniente Capurro, Pilar, Ñeembucú, Paraguay Fundación Para La Tierra, Centro IDEAL Pilar Paraguay

**Keywords:** Amphipoda, conservation, *Hyalella*, new species description, Paraguay, taxonomy

## Abstract

The freshwater amphipod genus *Hyalella* Smith, 1874 is widely distributed in the Neotropics, with several biogeographically restricted species and a high cryptic diversity throughout South America. Tens of species of *Hyalella* have been documented from nearby Brazil and Argentina, but no systematic record of the genus exists for Paraguay. Here we describe two new species of *Hyalella*: *H.mboitui***sp. nov.** and *H.julia***sp. nov.** from the Ñeembucú wetlands of southwestern Paraguay. *Hyalellamboitui***sp. nov.** and *H.julia***sp. nov.** are characterised by a dorsally smooth body, pigmented eyes, uropod 1 endopod with a curved seta, the dorsal margin of uropod 3 ramus without setae, and uropod 3 peduncle longer than wide and with six setae apically. The two species are distinguished by their diagnostic mouthparts, with a median serrated edge on the lacinia mobilis in *H.mboitui***sp. nov.** and two elongated lateral denticles with a serrated edge in *H.julia***sp. nov.**, and by the presence of a pronounced cup for the dactylus on gnathopod 2 in *H.mboitui***sp. nov**. In addition, they show differences in the number of articles on antennae 1 and 2, in the relative length of the pereiopods, and in the numbers and types of setae on their gnathopods and uropods 1–3. *Hyalellamboitui***sp. nov.** and *H.julia***sp. nov.** represent the first taxonomically documented occurrence of Paraguayan freshwater amphipods. These new taxa attest to the largely unmapped species richness of freshwater invertebrates in the Humid Chaco of Paraguay. This potential biodiversity hotspot is currently under threat from land conversion, highlighting the need for more systematic studies and effective conservation of the local invertebrate biodiversity.

## ﻿Introduction

Amphipods are a diverse clade of peracaridan crustaceans inhabiting both marine and freshwater environments, where they represent an ecologically and taxonomically significant component of the planktonic and benthic invertebrate fauna (Thomas 1993; Ishikawa and Urabe 2002). The genus *Hyalella* Smith, 1874 is endemic to the Americas and among the most widely distributed freshwater amphipods in the New World, ranging from southern Canada to Patagonia (Bueno et al. 2014; Damborenea et al. 2020; Reis et al. 2023). This diverse genus comprises more than 100 described species, and over 80 of them are endemic to South America (Marrón-Becerra and Hermoso-Salazar 2023; Marrón-Becerra et al. 2023; Peralta and Verónica 2023; Reis et al. 2023; Tomikawa et al. 2023). In particular, numerous species of the genus have been reported from Argentina, and southern Brazil, which holds the highest diversity of any single country (González et al. 2006; Talhaferro et al. 2021a, b; Reis et al. 2023). However, occurrences of this genus have not been systematically documented in their neighbouring country, Paraguay. Recent taxonomic and genetic studies of *Hyalella* suggest that the full extent of its diversity and distribution is vastly underestimated (Limberger et al. 2021; Talhaferro et al. 2021a; Waller et al. 2022). Therefore, reported discrepancies in regional taxonomic richness may be largely due to limited sampling (Reis et al. 2020, 2023).

This knowledge gap has potentially broad-ranging repercussions for conservation and habitat management. Amphipods, including *Hyalella*, sustain key links in matter and energy transfers in freshwater ecosystems, where they act as ecologically abundant grazers and detritivores, provide hosts for diverse micro- and macro-parasites, and serve as important prey items for both vertebrate and invertebrate predators (Duffy and Hay 2000; Castiglioni and Bond-Buckup 2008; Giari et al. 2020; Streck-Marx and Castiglioni 2020). Members of *Hyalella* support these critical ecosystem functions across diverse habitats, from hypogean waters to wetlands, from sea level up to above 4000 metres of elevation and inhabit both benthic sediments and a range of aquatic macrophytes (Castiglioni and Bond-Buckup 2008; Limberger et al. 2021; Zapelloni et al. 2021).

Here we describe two new species of *Hyalella* from the Ñeembucú wetlands, part of the Humid Chaco ecoregion of southwestern Paraguay (Mereles et al. 2020). These taxa represent the first scientifically documented occurrence of *Hyalella* in the country and present distinctive limb and mouthpart morphologies not reported for other congeneric species. The taxonomic distinctiveness and ecology of the two species are discussed considering the potential conservation threats to the freshwater habitats of the Ñeembucú region.

## ﻿Materials and methods

Freshwater invertebrate specimens were sampled from September 8, 2021 to June 5, 2023, as part of an environmental impact assessment led by Fundación Para La Tierra (PLT) under contract from the Ministry of Public Works and Communications (Ministerio de Obras Públicas y Comunicaciones, MOPC). Five field sites in total were sampled in and near the city of Pilar, Ñeembucú Department (Paraguay): Yegros Paso (26°51'51"S, 58°16'11"W), San Lorenzo (26°52'35"S, 58°18'40"W), Costanera (26°50'52"S, 58°18'51"W), Ring Road (26°52'31"S, 58°14'59"W) and Laguna Gadea (26°50'9"S, 58°18'46"W). Samples were collected using a Seine net in 100 m transects, fragmented into 10 mini-transects of 10 m each. Upon completing the 100 m transect, investigators returned to the beginning, completing as many transects as allowed in a 2-hour period of continuous sampling. There were two 2-hour periods at each site (a total of 4 hours per site), between 7:00 and 9:00, and again between 15:00 and 17:00. This was repeated every three months for 2021–2023. The invertebrate specimens collected were all placed in jars with 70% ethanol and transferred to the PLT laboratory [Centro IDEAL (Investigation, Development, Environmental Education and Leadership), Pilar] for examination and identified to the lowest taxonomic level permitted by the available literature.

Measurements for the two new species were taken under an AmScope Trinocular Stereo Zoom Microscope 3.5×–90× magnification with a millimetric scale. Representative specimens (male paratypes and female allotypes) were dissected using a scalpel, pincers and fine needles, and mounted on permanent slides for storage and drawing under an OMAX 40×–2000× LED Microscope with built-in camera. Our description follows the setal terminology of Zimmer et al. (2009).

Type specimens are stored in the Scientific Collection of PLT (Colección Científica de PLT, CCPLT) at Centro IDEAL in Pilar, Ñeembucú Department (Paraguay).

## ﻿Taxonomy

**Order Amphipoda Latreille, 1816**

**Family Dogielinotidae Gurjanova, 1953**

**Subfamily Hyalellinae Bulycheva, 1957**

**Genus *Hyalella* Smith, 1874**

### ﻿ Hyalella mboitui sp. nov.

Taxon classificationAnimaliaAmphipodaHyalellidae

7512E1A5-487D-5E16-9F6B-1C21F077D442

https://zoobank.org/B7930020-036E-457F-AEDF-26DE31C2B599

#### Type material.

**Figure 1.**
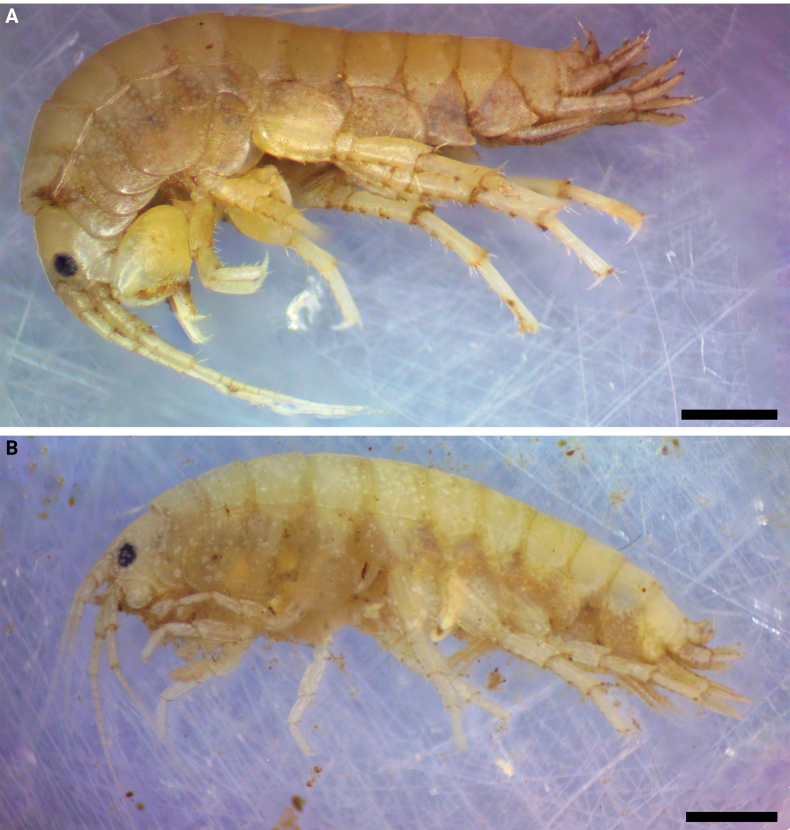
*Hyalellamboitui* sp. nov., Department of Ñeembucú, Paraguay **A** holotype, male **B** allotype, female. Scale bars: 1 mm.

**Figure 2.**
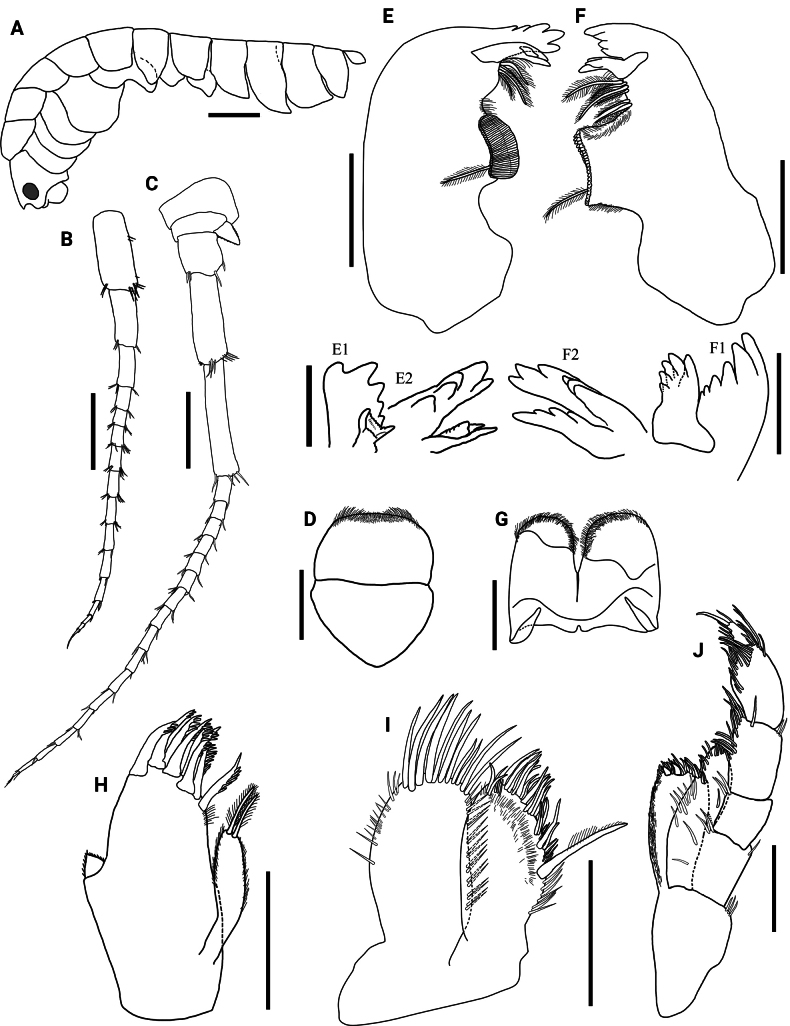
*Hyalellamboitui* sp. nov., Department of Ñeembucú, Paraguay. Paratype, male **A** habitus **B** antenna 1 **C** antenna 2 **D** upper lip **E** left mandible, with detail of lacinia and incisor rotated anticlockwise (**E1**) and clockwise (**E2**) **F** right mandible, with detail of lacinia and incisor rotated anticlockwise (**F1**) and clockwise (**F2**) **G** lower lip **H** maxilla 1 **I** maxilla 2 **J** maxilliped. Scale bars: 1 mm (**A**); 0.5 mm (**B, C**); 0.2 mm (**D–J**); 0.1 mm (**E1, E2, F1, F2**).

***Holotype***, male (Fig. 1A), total body length = 8.29 mm, head length = 0.89 mm (CIPLT-O-38); Allotype female (Fig. 2A), total body length = 7.10 mm, head length = 0.70 mm (CIPLT-O-38). Paraguay, Department of Ñeembucú, Pilar, Ring Road field locality (26°52'31"S, 58°14'59"W), September, 08, 2021. ***Paratypes*.** 43 males, 54 females, Ring Road (CIPLT-O-37; 26°52'31"S, 58°14'59"W) and San Lorenzo (CIPTL-O-39; 26°52'35"S, 58°18'40"W) field localities.

#### Type locality.

Paraguay, Department of Ñeembucú, Pilar, Ring Road field locality (26°52'31"S, 58°14'59"W).

#### Diagnosis.

Flagella of antennae 1 and 2 with 13–14 and 16–17 articles, respectively. Left mandible incisor toothed, 5-denticulate; left lacinia mobilis multi-denticulate, with median serrated surface and two prominent elongated denticles laterally. Gnathopod 1 propodus subtriangular, without triangular space between propodus and dactylus, with papposerrate setae on disto-anterior corner. Gnathopod 2 propodus with papposerrate setae on disto-posterior margin, with palm with pronounced cup for dactylus. Pereopod 5 shorter than other pereopods. Uropod 1 endopod with a curved seta. Uropod 3 ramus dorsal margin without setae. Uropod 3 peduncle with two cuspidate setae and four simple setae apically. Uropod 3 peduncle longer than wide (rectangular).

#### Description.

**Figure 3.**
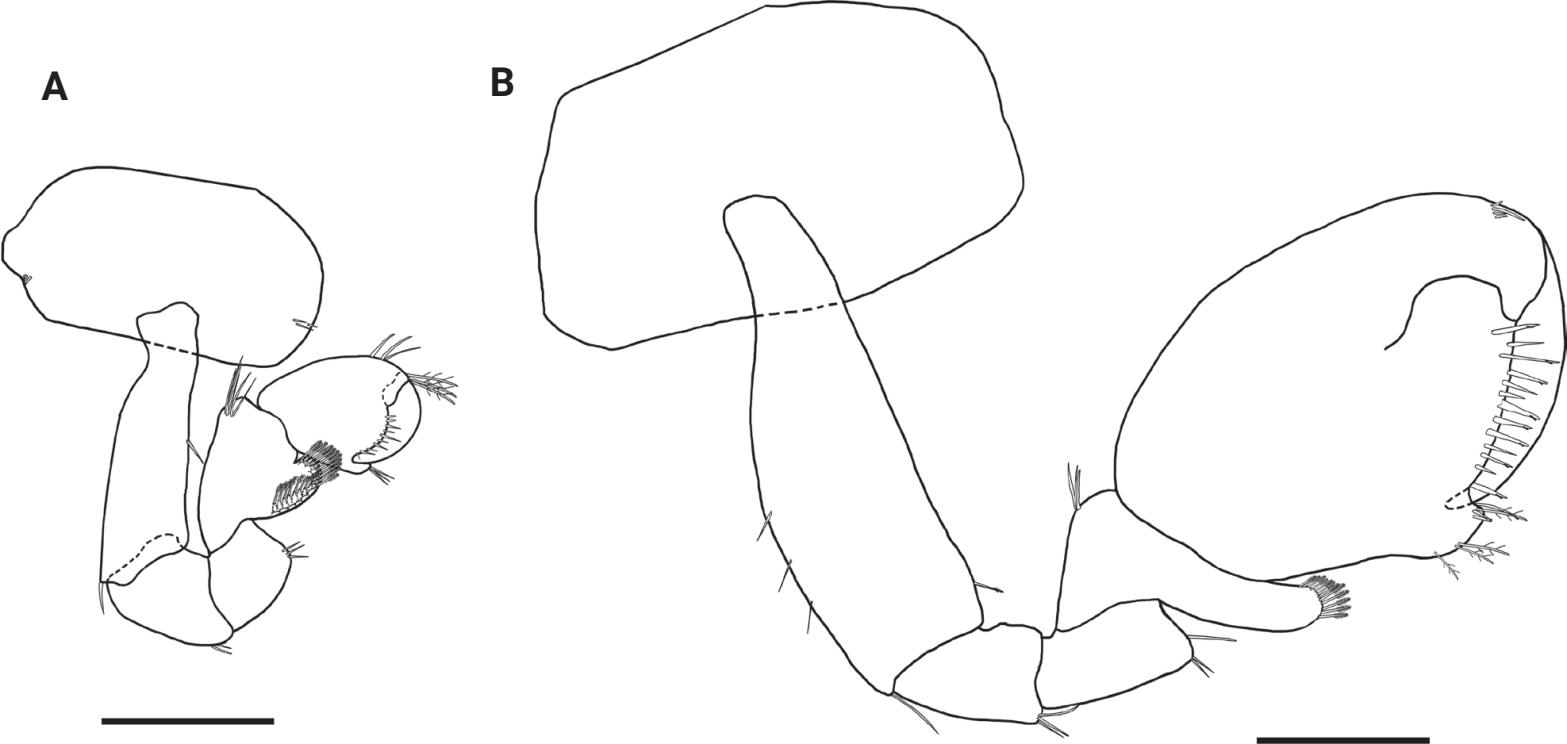
*Hyalellamboitui* sp. nov. Paratype, male **A** gnathopod 1 **B** gnathopod 2. Scale bars: 0.5 mm.

**Figure 4.**
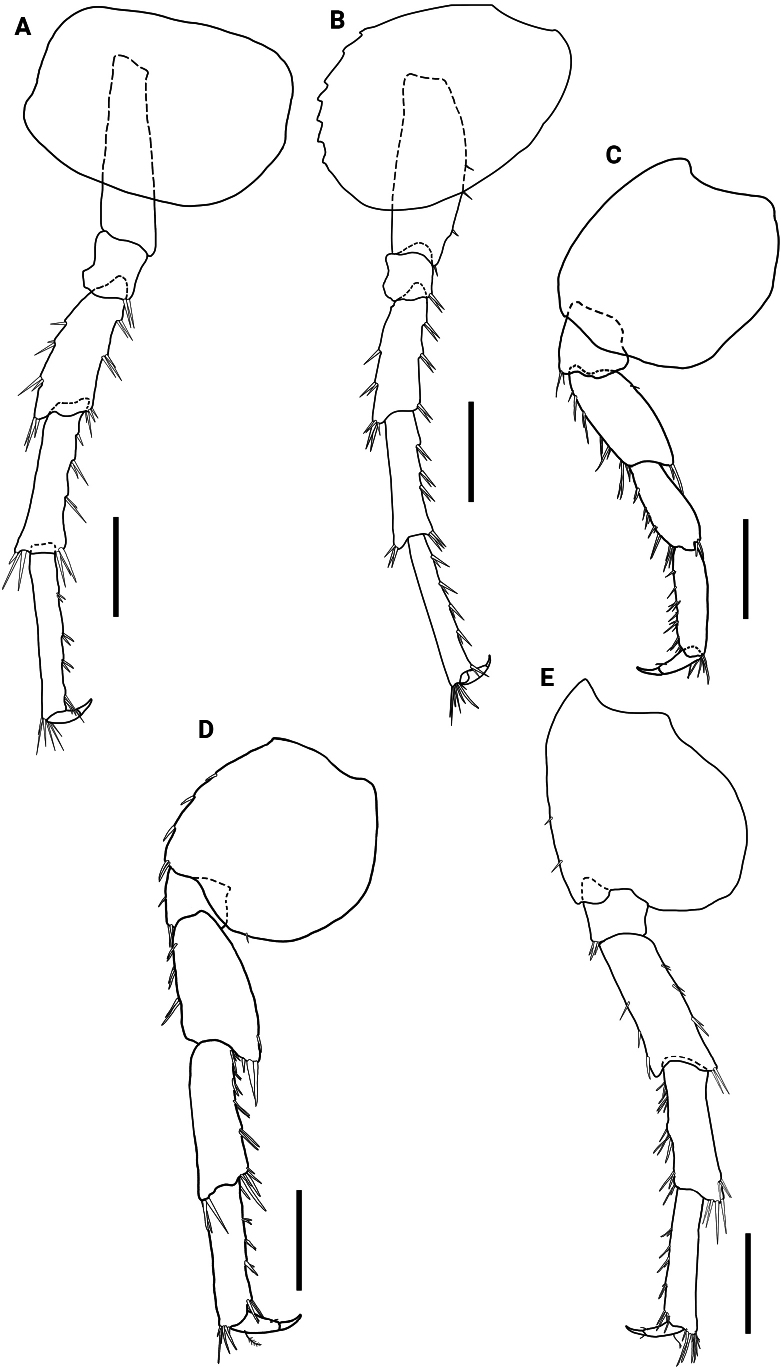
*Hyalellamboitui* sp. nov. Paratype, male **A** pereopod 3 **B** pereopod 4 **C** pereopod 5 **D** pereopod 6 **E** pereopod 7. Scale bars: 0.5 mm.

**Figure 5.**
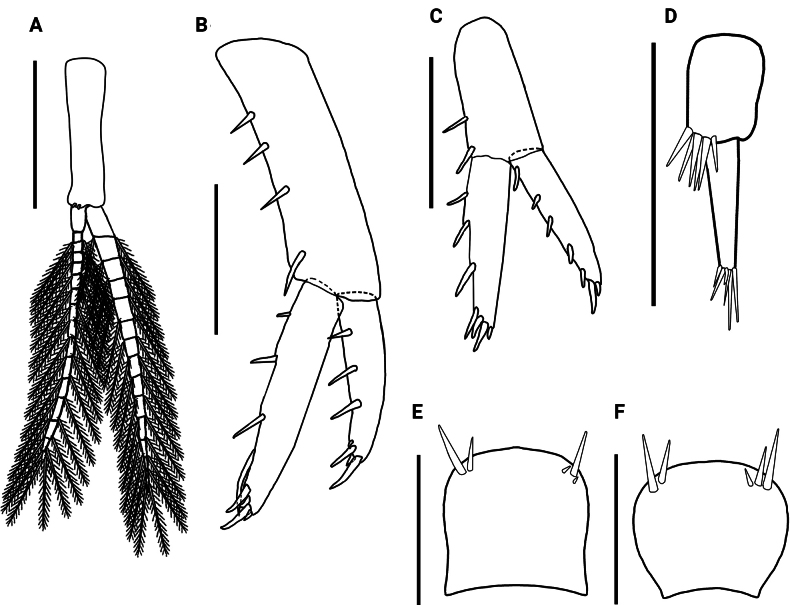
*Hyalellamboitui* sp. nov. Paratype, male. **A** pleopod **B** uropod 1 **C** uropod 2 **D** uropod 3 **E** male telson **F** paratype, female telson. Scale bars: 0.5 mm (**A–D**); 0.2 mm (**E, F**).

**Male** (Figs 1–5). Mean total body length: 7.98 mm; mean head length: 0.84 mm (*N* = 44). Body surface smooth. Epimeral plates not acuminate. Head smaller than first two thoracic segments, typically gammaridean, rostrum absent. Eyes pigmented, rounded, located between insertion of antennae 1 and 2 (Fig. 2A).

Antenna 1 about 2.2× shorter than body length, 1.4× shorter than antenna 2, 1.8× longer than peduncle of antenna 2; peduncle 1.1× longer than head length; flagellum with 13–14 articles, 1.5× longer than peduncle; aesthetascs occurring distally after article 4 (Fig. 2B).

Antenna 2 about 1.5× shorter than body length; peduncle 1.6× longer than head length; articles 1 to 3 with several simple setae on distal margin; flagellum 1.7× longer than peduncle, with 16–17 articles, with basal article elongated, with several simple setae on distal margins, and with four simple setae apically (Fig. 2C).

Basic amphipodan mandibles (sensu Watling 1993), without palp; left incisor toothed, 5-denticulate; left lacinia mobilis multi-denticulate, with medial surface with multiple small serrated denticles and two prominent elongated denticles laterally, setal row with five papposerrate setae, with setules, molar process large and cylindrical, triturative, with one accessory seta (Fig. 2E). Right mandible incisor 6-denticulate; lacinia 5-denticulate, setal row with six papposerrate setae, with setules.

Upper lip distal margin truncate; distal border covered by setules on ventral and dorsal faces (Fig. 2D). Lower lip outer lobes rounded, not notched, with several setules on dorsal and ventral faces (Fig. 2G).

Maxilla 1 inner plate slender, 1.8× shorter than outer plate, with two apical papposerrate setae and several setules laterally. Outer plate with nine serrate setae and several setules (Fig. 2H). Palp short, uniarticulate, 1.0× longer than wide, with apical and lateral setules, reaching less than half distance between base of palp and base of setae on outer plate.

Maxilla 2 inner and outer plates subequal in length and width. Inner plate with one papposerrate seta and several simple and serrate setae apically, and several setules on inner face; outer plate with several simple setae on apex and margin, longest apically (Fig. 2I).

Maxilliped inner plate 2.0× longer than wide, apically truncated, with two cuspidate setae, several simple setae apically, and several setules on inner margin, comb-scales absent; outer plate approximately 1.3× longer than inner plate, apically rounded, with several apical and lateral simple setae, comb-scales absent; palp approximately 2.1× longer than inner plate, with four articles; article 1 1.1× longer than wide, inner margin with few simple setae; article 2 1.5× longer than wide, inner margin with several simple setae, outer margin with few simple setae; article 3 1.8× longer than wide, inner and outer margins with several long simple setae; article 4 unguiform, 3.0× shorter than third article, 1.6× longer than wide, inner margin with several long simple setae, with distal simple seta, with distal nail and comb-scales absent (Fig. 2J).

Gnathopod 1 subchelate; coxal plate 2.1× wider than long, with several simple setae on anterior and posterior margins; basis with one simple seta on anterior margin and one on disto-posterior corner; ischium with few simple setae on disto-posterior corner; merus with few simple setae on posterior margin; carpus 1.3× longer than wide, 1.1× longer and wider than propodus, posterior lobe produced and forming scoop-like structure, pectinate margin with several serrate setae, comb-scales and polygonal pattern; propodus 1.3× longer than wide, hammer-shaped, with several simple setae on anterior margin, with several papposerrate setae on disto-anterior corner; palm slope oblique, with several simple setae, margin convex, disto-posterior corner with long simple setae, and with a pronounced cup for dactylus; dactylus claw-like, congruent with palm, without comb-scales (Fig. 3A).

Gnathopod 2 subchelate; coxal plate 1.6× wider than long; basis with few simple setae on posterior margin and one serrate seta on disto-anterior margin; ischium and merus with few simple setae on posterior margin; carpus 1.8× wider than long, posterior lobe slim, produced between merus and propodus, margin pectinate with several serrate setae; propodus ovate, 1.4× longer than wide, comb-scales absent; palm subequal to posterior margin of propodus, slope oblique, margin convex, slightly irregular, with several simple setae and cuspidate setae with accessory seta; disto-posterior corner with two small cuspidate setae and several papposerrate setae, and with a pronounced cup for dactylus; dactylus claw-like, congruent with palm, without comb-scales (Fig. 3B).

Pereopods 3 to 7 simple. Pereopod 4 (Fig. 4B) with several simple setae on basis posterior margin; pereopods 3 (Fig. 4A) and 4 with several simple setae on merus and carpus posterior margins, with several simple and cuspidate setae on propodus posterior margins; dactylus approximately 3.5× shorter than propodus, in both. Pereopods 5, 6 and 7 (Fig. 4C–E) with merus, carpus and propodus posterior margins with several cuspidate and simple, dactylus 1.8×, 2.1× and 2.2× shorter than propodus, respectively, unguiform, with plumose seta dorsally on pereopods 6 and 7. Pereopod 3 and 4 of similar sizes; pereopod 5 smaller than others; pereopod 6 slightly shorter than pereopod 7.

Pleopods not modified, biramous, elongated; peduncle 4.0× longer than wide, 1.7× mean size of rami, with coupling spines distally; both rami multi-annulated, longer than peduncle, with articles decreasing in size distally, with several plumose setae (Fig. 5A).

Uropod 1 1.5× longer than uropod 2; peduncle 1.1× longer than longest ramus, with four cuspidate setae; inner ramus 1.4× longer than outer ramus, 4.9× longer than wide, with three dorsal cuspidate setae, with one long curved seta and four cuspidate setae apically; outer ramus with three dorsal cuspidate setae and four cuspidate setae apically (Fig. 5B).

Uropod 2 1.5× shorter than uropod 1; peduncle rectangular, subequal in length to outer ramus and 1.2× shorter than inner ramus, 2.0× wider than outer ramus and 1.5× than inner ramus, with two cuspidate setae; inner ramus slightly longer than outer ramus, with four cuspidate setae dorsally and three cuspidate setae apically; outer ramus with three cuspidate setae dorsally and four cuspidate setae apically (Fig. 5C).

Uropod 3 (Fig. 5D) 2.2× shorter than peduncle of uropod 1 and 1.1× than peduncle of uropod 2; peduncle 1.5× longer than wide, 3.7× wider than ramus, with six apical long cuspidate setae; inner ramus absent; outer ramus uniarticulate, 1.2× longer than peduncle, with two cuspidate and four simple setae apically.

Telson entire, 1.1× longer than wide, apically rounded, without setae laterally, with five apical cuspidate setae (Fig. 5E).

Coxal gills sac-like present on pereonites 3 to 6; sternal gills tubular and present on pereonites 3 to 7.

**Figure 6.**
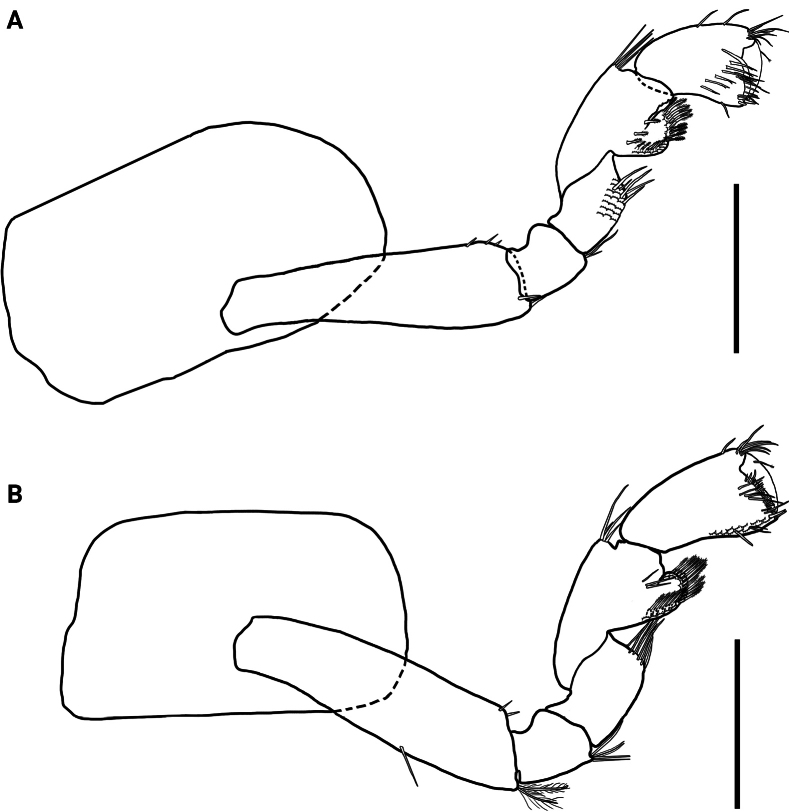
*Hyalellamboitui* sp. nov. Paratype, female. **A** gnathopod 1 **B** gnathopod 2. Scale bars: 0.5 mm.

**Female** (Figs 1B, 5E, 6). Mean total body length: 6.23 mm; mean head length: 0.62 mm (*N* = 55).

Antennae similar in shape to male. Antenna 1 flagellum with 10–11 articles. Antenna 2 flagellum with 16–17 articles.

Gnathopod 1 (Fig. 6A) similar to male gnathopod 1 in size but different in shape; basis with few simple setae on disto-anterior and disto-posterior margins; ischium with few simple setae on disto-posterior margin; merus with several simple setae and comb-scales on posterior margin; carpus 1.7× longer than wide, 1.3× longer and 1.2× wider than propodus, with several simple setae on disto-anterior corner, posterior lobe produced and forming scoop-like structure, with pectinate margin, with comb-scales, with several serrate setae and polygonal pattern, and with three serrate setae on inner margin; propodus 1.6× longer than wide, hammer-shaped, inner margin with six simple setae with accessory setae, dorsal margin with two simple setae, disto-anterior corner with several simple setae; palm 1.4× shorter than posterior margin of propodus, slope transverse, margin slightly irregular, with several simple setae, with few simple setae and two cuspidate setae on disto-posterior corner; dactylus claw-like, with one plumose seta dorsally.

Gnathopod 2 (Fig. 6B) similar in size and shape to gnathopod 1; basis with one simple seta on posterior margin, with few simple setae on disto-anterior corner, and two pappose setae on disto-posterior corner; ischium with several simple setae on disto-posterior corner; merus with several simple setae on posterior margin; carpus 1.5× longer than wide, 1.0× longer and 1.3× wider than propodus, with several simple setae on disto-anterior corner, posterior lobe produced and forming scoop-like structure with pectinate margin, with comb-scales, with several serrate setae and polygonal pattern, inner margin with few simple setae; propodus longer than wide, hammer-shaped, with comb-scales and several simple setae on disto-posterior margin, and several simple setae on disto-anterior corner; inner face with several simple setae; palm 1.9× shorter than posterior margin of propodus, slope oblique, margin slightly concave, with several simple setae, disto-posterior corner with two cuspidate setae; dactylus claw-like, with one plumose seta dorsally.

Telson approximately as long as wide, with more convex lateral margins than in male, and with five cuspidate setae apically (Fig. 5F).

Uropod 1 similar in size and shape to male uropod 1, except for absence of curved seta.

#### Habitat.

Freshwater, epigean.

#### Distribution.

Paraguay, Department of Ñeembucú, Pilar. Field localities of Ring Road (26°52'31"S, 58°14'59"W) and San Lorenzo (26°52'35"S, 58°18'40"W).

#### Etymology.

In reference to Mbói Tu’i, one of the seven legendary monsters of Guaraní mythology and protector of wetlands and aquatic life. The species is named in Guaraní in honour of it being an endemic Paraguayan species.

### ﻿ Hyalella julia sp. nov.

Taxon classificationAnimaliaAmphipodaHyalellidae

6F402ABB-05D3-519F-B17F-8AABB2DEB423

https://zoobank.org/4F2CE36F-B2A1-47AB-A812-ED4CC0D6D4A7

#### Type material.

**Figure 7.**
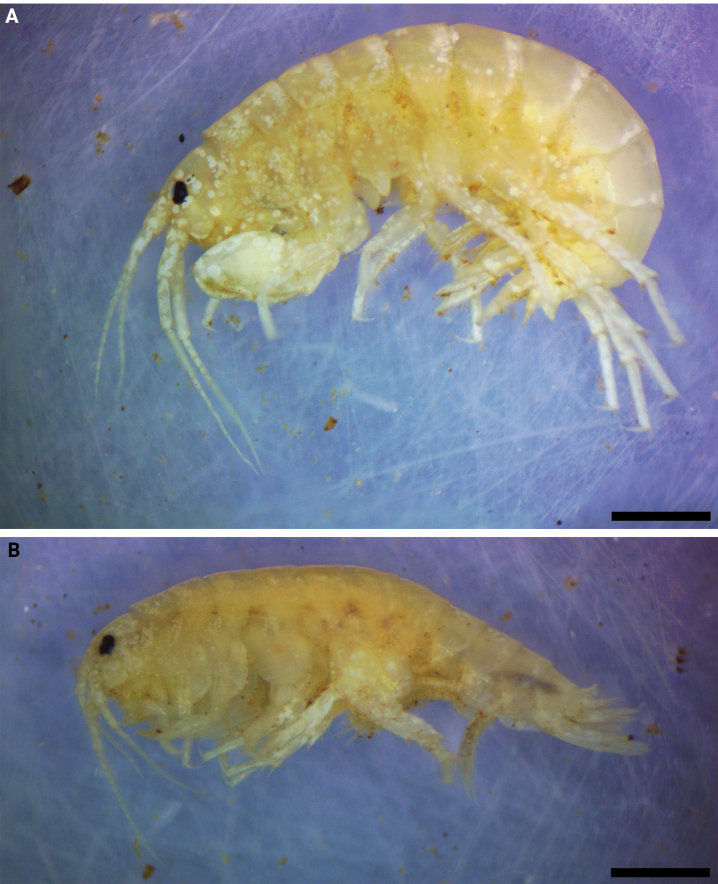
*Hyalellajulia* sp. nov., Department of Ñeembucú, Paraguay **A** holotype, male **B** allotype, female. Scale bars: 1 mm.

***Holotype***, male (Fig. 7A), total body length = 8.81 mm, head length = 0.78 mm (CIPLT-O-40); Allotype female (Fig. 7B), total body length = 5.44 mm, head length = 0.42 mm (CIPLT-O-40). Paraguay, Department of Ñeembucú, Pilar, Yegros Paso field locality (26°51'51"S, 58°16'11"W), September, 06, 2021. ***Paratypes*.** 9 males, 20 females (CIPLT-O-40), Yegros Paso field locality (26°51'51"S, 58°16'11"W).

#### Type locality.

Paraguay, Department of Ñeembucú, Pilar, Yegros Paso field locality (26°51'51"S, 58°16'11"W).

#### Diagnosis.

Flagella of antennae 1 and 2 with 10–11 and 13–14 articles, respectively. Left mandible incisor toothed, 4-denticulate; left lacinia mobilis 3-denticulate, with short median denticle and two prominent elongated denticles with serrated margin laterally. Gnathopod 2 propodus with palm lacking pronounced cup for dactylus, without papposerrate setae, with cuspidate setae with accessory setae on disto-posterior corner. Pereopod 5 slightly longer than other pereopods. Uropod 1 endopod with a curved seta. Uropod 3 ramus dorsal margin without setae. Uropod 3 peduncle with six simple setae apically. Uropod 3 peduncle longer than wide (rectangular).

#### Description.

**Figure 8.**
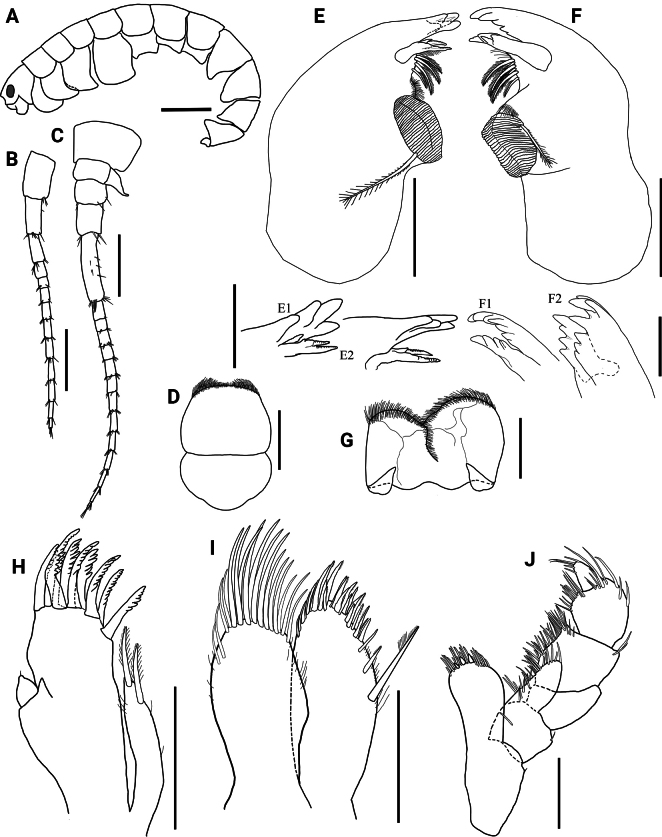
*Hyalellajulia* sp. nov., Department of Ñeembucú, Paraguay. Paratype, male **A** habitus **B** antenna 1 **C** antenna 2 **D** upper lip **E** left mandible with detail of lacinia and incisor rotated anticlockwise (**E1**) and clockwise (**E2**) **F** right mandible, with detail of lacinia and incisor rotated anticlockwise (**F1**) and clockwise (**F2**) **G** lower lip **H** maxilla 1 **I** maxilla 2 **J** maxilliped. Scale bars: 1 mm (**A**); 0.5 mm (**B, C**); 0.2 mm (**D–J**); 0.1 mm (**E1, E2, F1, F2**).

**Figure 9.**
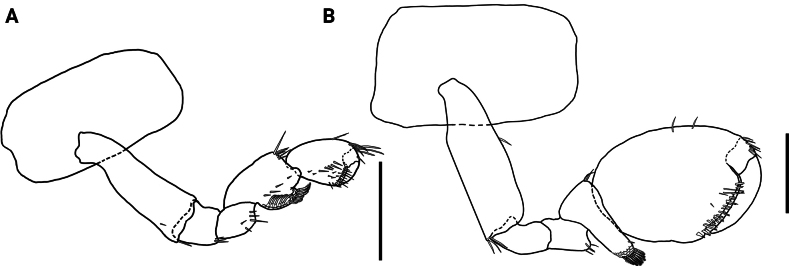
*Hyalellajulia* sp. nov. Paratype, male **A** gnathopod 1 **B** gnathopod 2. Scale bars: 0.5 mm.

**Figure 10.**
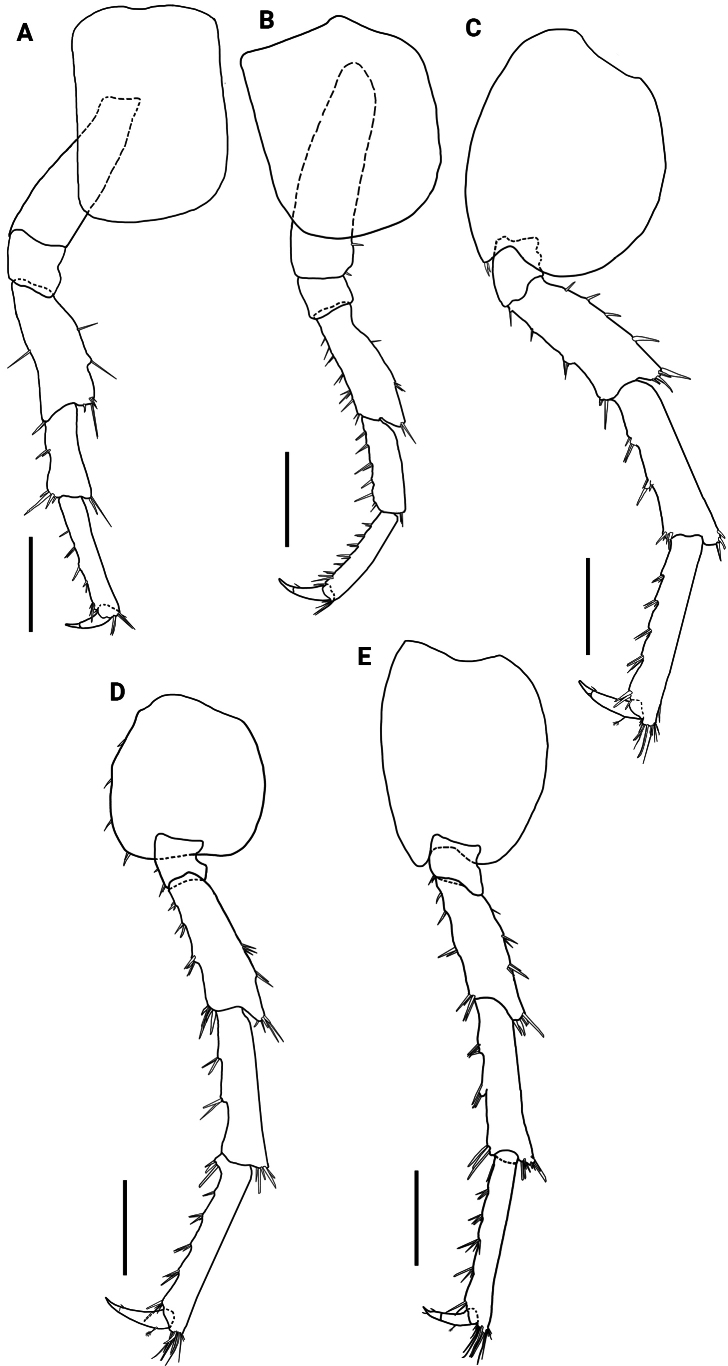
*Hyalellajulia* sp. nov. Paratype, male **A** pereopod 3 **B** pereopod 4 **C** pereopod 5 **D** pereopod 6 **E** pereopod 7. Scale bars: 0.5 mm.

**Figure 11.**
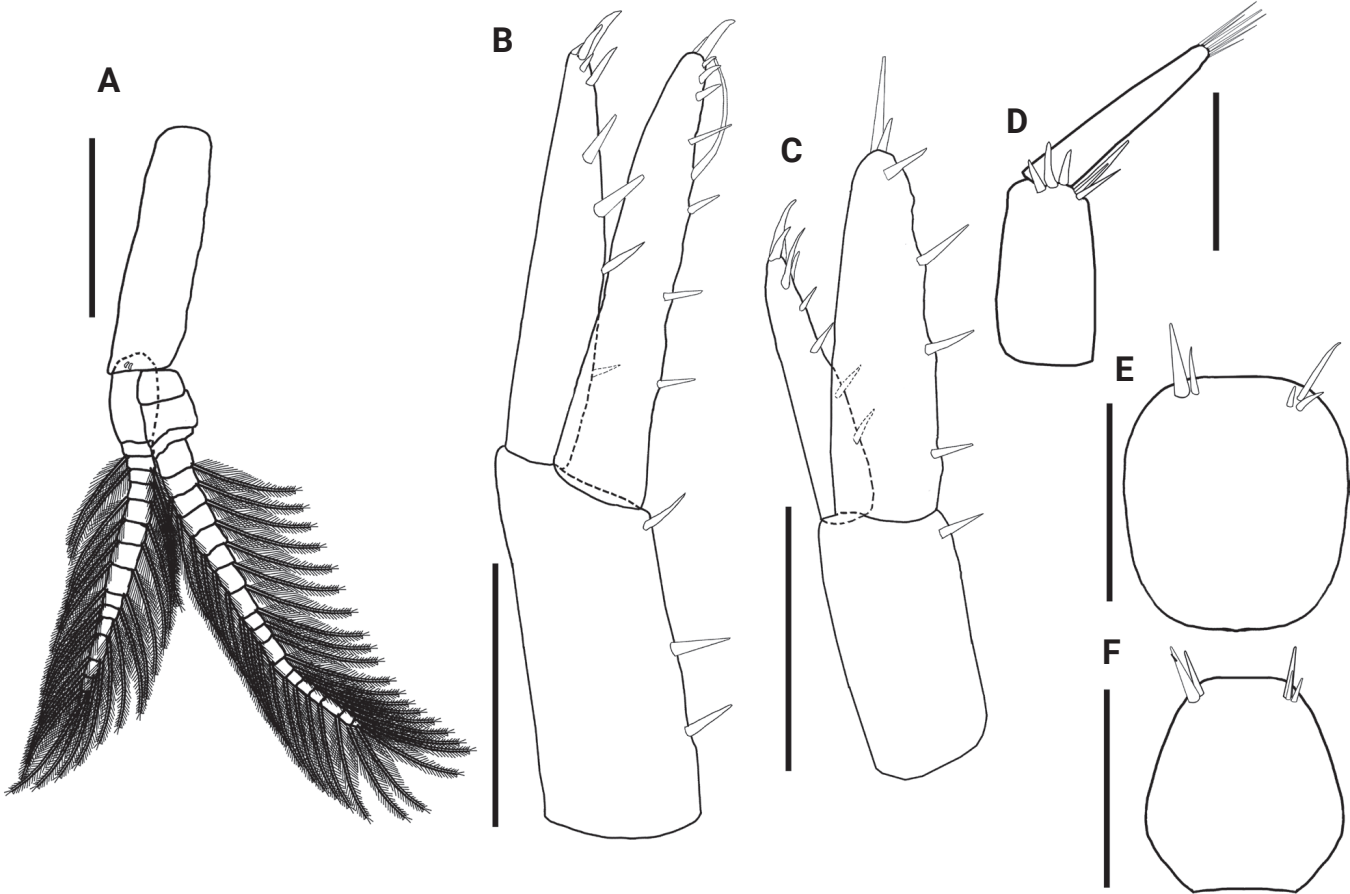
*Hyalellajulia* sp. nov. Paratype, male **A** pleopod **B** uropod 1 **C** uropod 2 **D** uropod 3 **E** male telson **F** paratype, female telson. Scale bars: 0.5 mm (**A–C**); 0.2 mm (**D–F**).

**Male** (Figs 7–11). Mean total body length: 7.24 mm; mean head length: 0.76 mm (*N* = 10). Body surface smooth. Epimeral plates not acuminate. Head smaller than first two thoracic segments, typically gammaridean, rostrum absent. Eyes pigmented, ovoid, located between insertion of antennae 1 and 2 (Fig. 8A).

Antenna 1 about 3.2× shorter than body length, 1.3× shorter than antenna 2, 2.2× longer than peduncle of antenna 2; peduncle not surpassing head length; flagellum with 10–11 articles, 2.1× longer than peduncle; aesthetascs occurring distally after article 4 (Fig. 8B).

Antenna 2 about half of body length; peduncle 1.1× longer than head; articles 1 to 3 with several simple setae on distal margin, article 3 with several simple setae on lateral margin; flagellum 2.4× longer than peduncle, with 13–14 articles, with basal article elongated; articles with several simple setae on distal margins; four simple setae apically (Fig. 8C).

Basic amphipodan mandibles (sensu Watling 1993), without palp; left incisor toothed, 4-denticulate; left lacinia mobilis 3-denticulate, with short median denticle, with two prominent elongated denticles with serrated upper margin laterally. Setal row with four papposerrate setae, molar process large and cylindrical, triturative, with one accessory seta (Fig. 8E). Right mandible incisor 7-denticulate; lacinia 4-denticulate, setal row with six papposerrate setae (Fig. 8F).

Upper lip distal margin rounded, covered by several setules on dorsal and ventral faces (Fig. 8D). Lower lip outer lobes rounded and distally notched, covered distally by several setules on dorsal and ventral faces (Fig. 8G).

Maxilla 1 inner plate slender, 1.4× shorter than outer plate, with two apical papposerrate setae and several setules laterally; outer plate with nine serrate setae (Fig. 8H). Palp short, uniarticulate, 1.2× longer than wide, with a distal setule, reaching less than half of distance between base of palp and base of setae on outer plate.

Maxilla 2 inner plate 1.1× longer than outer plate; inner plate with one papposerrate seta and several simple and serrate setae apically, with several setules laterally; outer plate with several simple setae, longest apically, with several setules laterally (Fig. 8I).

Maxilliped inner plate 1.7× longer than wide, apically truncated, with three apical cuspidate setae and several simple setae, without comb-scales; outer plate 1.3× longer than inner plate, apically rounded, with several apical and lateral simple setae; palp 2.3× longer than inner plate, 1.7× longer than outer plate, with four articles; article 1 1.3× longer than wide, with strongly concave distal margin; article 2 1.0× longer than wide, with inner, outer, and distal margins with several long simple setae; article 3 1.2× longer than wide, with inner and outer margins with several simple setae; article 4 unguiform, 1.5× longer than wide, 2.0× shorter than third article, with distal simple setae, with distal nail and comb-scales absent (Fig. 8J).

Gnathopod 1 subchelate; coxal plate 1.9× wider than long; basis with one simple seta on inner margin and one on disto-posterior corner, ischium with few simple setae on disto-posterior corner; merus with several simple setae on posterior margin; carpus 1.5× longer than wide, 1.2× longer and 1.2× wider than propodus, with several simple setae on disto-anterior corner, some with accessory seta, with few simple setae on inner margin, with posterior lobe folded to form scoop-like structure, with pectinate margin with comb-scales, several serrate setae and polygonal pattern; propodus 1.6× longer than wide, hammer-shaped, with simple seta with accessory seta on anterior margin, with inner margin with several simple setae, with several long simple setae on disto-anterior corner; palm slope transverse, margin slightly concave, with many simple setae, with disto-posterior corner with cuspidate seta with accessory seta; dactylus claw-like, congruent with palm, without comb-scales (Fig. 9A). Microtrichs present on propodus.

Gnathopod 2 subchelate; coxal plate 1.8× wider than long; basis with one simple seta on anterior margin and several simple setae on disto-posterior margin; merus with several simple setae on posterior margin; carpus 2.0× wider than long, with one simple seta on inner margin and two on disto-anterior, with posterior lobe slim produced between merus and propodus, with posterior margin pectinate, with several serrate setae and comb-scales; propodus ovate, 1.4× longer than wide, with two simple setae on anterior margin; palm subequal to posterior margin of propodus, slope oblique, margin convex, with several long and short simple setae with accessory setae; disto-posterior corner with two cuspidate setae with accessory setae; very shallow cup for dactylus; dactylus claw-like, congruent with palm, without comb-scales (Fig. 9B).

Pereopods 3 to 7 simple. Pereopods 3 and 4 (Fig. 10A, B) with posterior margins of merus and carpus with several simple and cuspidate setae; propodus posterior margin with several simple and cuspidate setae; dactylus 2.6× and 1.9× shorter than propodus in pereopods 3 and 4, respectively, unguiform. Pereopods 5 to 7 (Fig. 10C–E) with posterior margins of merus, carpus and propodus with several cuspidate and simple setae; dactylus 2.7×, 2.6×, and 3.0× shorter than propodus, respectively, unguiform, with a plumose seta dorsally. Pereopod 3 and 4 of similar sizes, shorter than pereopods 5–7; pereopods 6 and 7 of similar length, pereopod 5 slightly longer than other pereopods.

Pleopods not modified, biramous, elongated; peduncle 4.0× longer than wide, 1.5× shorter than mean size of rami, with coupling spines distally; both rami multi-annulated, longer than peduncle; articles decreasing in size distally in both rami; both rami with several plumose setae (Fig. 11A).

Uropod 1 1.3× longer than uropod 2; peduncle 1.1× shorter than outer ramus and 1.2× shorter than inner ramus, with three cuspidate setae; inner ramus 1.1× longer than outer ramus, with four cuspidate setae dorsally, and one long curved seta and five cuspidate setae apically; outer ramus with four cuspidate setae dorsally and five cuspidate setae apically (Fig. 11B).

Uropod 2 1.3× shorter than uropod 1; peduncle rectangular, 1.0× shorter than outer ramus and 1.3× than inner ramus, 2.4× wider than outer ramus and 1.3× than inner ramus, with one cuspidate seta dorsally; inner ramus 1.3× longer than outer ramus, with three cuspidate setae dorsally and three cuspidate setae apically; outer ramus with four cuspidate setae dorsally and four cuspidate setae apically (Fig. 11C).

Uropod 3 (Fig. 11D) 1.6× shorter than peduncle of uropod 1 and 1.1× than peduncle of uropod 2; peduncle 1.9× longer than wide, 2.0× wider than ramus, with six cuspidate setae apically; inner ramus absent; outer ramus uniarticulate, subequal in length to peduncle, with six simple setae apically.

Telson entire, 1.1× longer than wide, with convex margins, and rounded apically, without setae laterally, and with five cuspidate setae apically (Fig. 2D).

Coxal gills sac-like present on pereonites 3 to 6; sternal gills tubular and present on pereonites 3 to 7.

**Figure 12.**
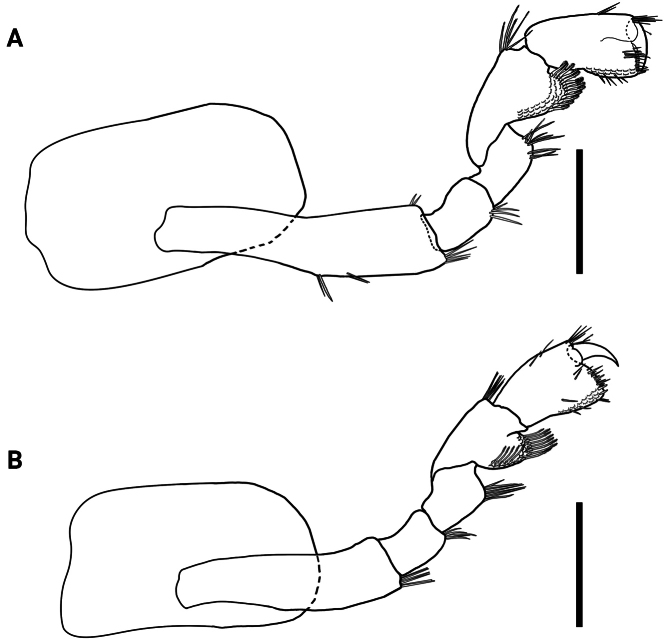
*Hyalellajulia* sp. nov. Paratype, female **A** gnathopod 1 **B** gnathopod 2. Scale bars: 0.5 mm.

**Female** (Figs 7B, 11E, 12). Mean total body length: 5.23 mm; mean head length: 0.46 mm (*N* = 21).

Antennae similar in shape to male. Antenna 1 flagellum with 10–11 articles. Antenna 2 flagellum with 11–12 articles.

Gnathopod 1 (Fig. 12A) slightly larger than male gnathopod 1 different from male gnathopod 1 in shape; basis with few simple setae on disto-anterior and posterior margins; ischium with several simple setae on disto-posterior margin; merus with several simple setae on posterior margin; carpus 1.6× longer than wide, with several serrate setae on disto-anterior corner, posterior lobe with pectinate margin, with comb-scales and one row of serrate setae; propodus 1.8× longer than wide, hammer-shaped; anterior margin with two simple setae, disto-anterior corner with several simple setae, posterior margin with several simple setae and comb-scales, inner margin with four simple setae; palm slope transverse, margin slightly irregular, slightly concave, with several simple setae, with few simple setae and two long cuspidate setae on disto-posterior corner; dactylus claw-like, with one plumose seta dorsally.

Gnathopod 2 (Fig. 12B) similar in size and shape to gnathopod 1; basis and ischium with several simple setae on disto-posterior corner; merus with several simple setae on posterior margin; carpus 1.3× longer than wide, with several simple setae on disto-anterior corner, posterior lobe produced and forming scoop-like structure, pectinate margin with comb-scales, several serrate setae and polygonal pattern; propodus 1.6× longer than wide, hammer-shaped, with comb-scales on disto-posterior margin, with few simple setae on anterior and posterior margins, with several long simple setae on disto-anterior corner, inner face with several simple setae; palm slope oblique, margin slightly irregular, with several simple setae, disto-posterior corner with two simple and two cuspidate setae; dactylus claw-like, with one plumose seta dorsally.

Telson subequal in length and width, with more convex lateral margins than in male, and with five cuspidate setae, one with accessory seta.

Uropod 1 similar in size and shape to male uropod 1, except for absence of curved seta.

#### Habitat.

Freshwater, epigean.

#### Distribution.

Paraguay, Department of Ñeembucú, Pilar. Field locality of Yegros Paso (26°51'51"S, 58°16'11"W).

#### Etymology.

In honour of the late Don Julio Rafael Contreras, for his seminal studies of Paraguayan biodiversity and generous support of Fundación Para La Tierra.

##### ﻿Taxonomic remarks

*Hyalellamboitui* sp. nov. and *H.julia* sp. nov. can be recognised as distinct species based on the taxonomic keys by Damborenea et al. (2020) and morphological differences from other recently described South American species (Reis et al. 2020; Jaume et al. 2021; Limberger et al. 2021; Rocha Penoni et al. 2021; Talhaferro et al. 2021a, b; Vernica et al. 2022; Waller et al. 2022; Peralta and Verónica 2023; Reis et al. 2023). Both *H.mboitui* and *H.julia* show a smooth body without dorsal or lateral processes or mucronations, have pigmented eyes, and lack setae on the dorsal margin of uropod 3. The presence of a curved seta on the ramus of male uropod 1 links both new taxa to a large cluster of South American species spanning Venezuela, Brazil, Chile, Argentina and Uruguay (Bastos-Pereira and Bueno 2012; Rodrigues et al. 2014; Damborenea et al. 2020; Talhaferro et al. 2021a).

Like *H.brasiliensis* Bousfield, 1996 from Paraná State (Brazil), both *H.mboitui* and *H.julia* lack plumose setae on their telson, but can be readily distinguished from this species by the number of setae on uropods 1 and 2 (Bousfield 1996; Talhaferro et al. 2021a, b). The presence of six apical setae on the rectangular peduncle of uropod 3 and its rectangular (longer than wide) shape in *H.mboitui* and *H.julia* are shared with the Argentinian taxa *H.pampeana* Cavalieri, 1968 and *H.bonariensis* Dos Santos, Bond-Buckup & Araujo, 2008, and with *H.gauchensis*Streck et al., 2017 from Rio Grande do Sul, Brazil (Damborenea et al. 2020). However, the two Paraguayan species lack the space between the dactylus and the margin of the propodus of male gnathopod 2 characteristics of *H.pampeana* (Dos Santos et al. 2008) and differ markedly from *H.bonariensis* in the pattern and distribution of setae and comb-scales on their limbs and telson, as well as in the numbers of denticles on the mandibular incisor and lacinia mobilis (Dos Santos et al. 2008). In addition, they differ from *H.gauchensis* in their mandibular morphology and setal cover of the telson and uropods (cf. Streck et al. 2017).

The two new Paraguayan taxa are also readily distinguishable from recently described *Hyalella* species from nearby Argentina (Peralta and Miranda 2019; Vernica and Alejandra 2022) and southern Brazil (Reis et al. 2020; Limberger et al. 2021; Rocha Penoni et al. 2021;Talhaferro et al. 2021a, b) by the number and type of setae on the telson and uropods 1 and 3 (Figs 5B, D, 11B, D). Their level of morphological differentiation also indicates that the new Paraguayan species cannot be subsumed under the South American *H.curvispina* Shoemaker, 1942 species complex, which appears to comprise significant cryptic diversity based on recent molecular marker analyses (Waller et al. 2022). Despite similarities in telson shape and the morphology and setal cover of maxillae and maxillipeds (Figs 2H–J, 8H–J; Shoemaker 1942; Grosso and Peralta 1999), *H.mboitui* and *H.julia* are distinguished from *H.curvispina* by their diagnostic mandibular dentition, the absence of a plumose seta on the dactyli of male gnathopods, the number of setae on the telson, and the shape and number of setae of the uropod 3 peduncle, which is wider than long in *H.curvispina* (Shoemaker 1942; Grosso and Peralta 1999; Damborenea et al. 2020) but not in *H.mboitui* and *H.julia* (Figs 5D, 11C).

Despite the geographical vicinity of their type locations, *H.mboitui* and *H.julia* are separated by clear morphological differences at the level of the gnathopods, uropods and mandibles, as well as by minor differences in the morphology and setal covers of their antennae, maxillae and maxillipeds. Antennae 1 and 2 have fewer articles in their flagellum in *H.julia* than in *H.mboitui*: *H.julia* has minimally 13 articles in antenna 1 and 16 in antenna 2, whereas *H.mboitui* has minimally 10 in antenna 1 and 13 in antenna 2. The mandibles of the two taxa differ in the number of incisor teeth, with 5 and 6 teeth present in the left mandibles of *H.mboitui* and *H.julia*, respectively, and 4 and 7 in their right mandibles. In addition, the left lacinia mobilis of *H.julia* lacks the distinctive median serrated surface of *H.mboitui*, and sports instead two prominent, elongated denticles with a serrated edge laterally. The maxillipeds of the two species differ in the number of cuspidate setae on the outer plate and in the shape of palp articles (Figs 2J, 8J). The male gnathopods of *H.julia* and *H.mboitui* differ in the number and type of setae: notably, papposerrate setae are absent in the disto-posterior corner of the gnathopod 2 propodues in *H.julia*, which shows instead two stout cuspidate setae with accessory setae (Fig. 9B, cf. Fig. 3B). Papposerrate setae are also present on gnathopod 1 in *H.mboitui*, but not *H.julia* (Fig. 9A, cf. Fig. 3A). In addition, *H.julia* lacks papposerrate setae on the disto-anterior margin of the gnathopod 1 propodus (Fig. 9A, cf. Fig. 3A). The propodi of female gnathopods are also more elongated and less subtriangular in *H.julia*, and differ in the presence and extent of their cover of comb-scales (Fig. 6, cf. Fig. 12). Pereopod 5 is the shortest pereopod in *H.mboitui*, but the longest in *H.julia*. Moreover, uropods 1, 2 and 3 in the two species differ in the number of cuspidate setae on their rami and peduncle (Figs 5B, C, 11B, C).

##### ﻿Habitat and conservation

**Figure 13.**
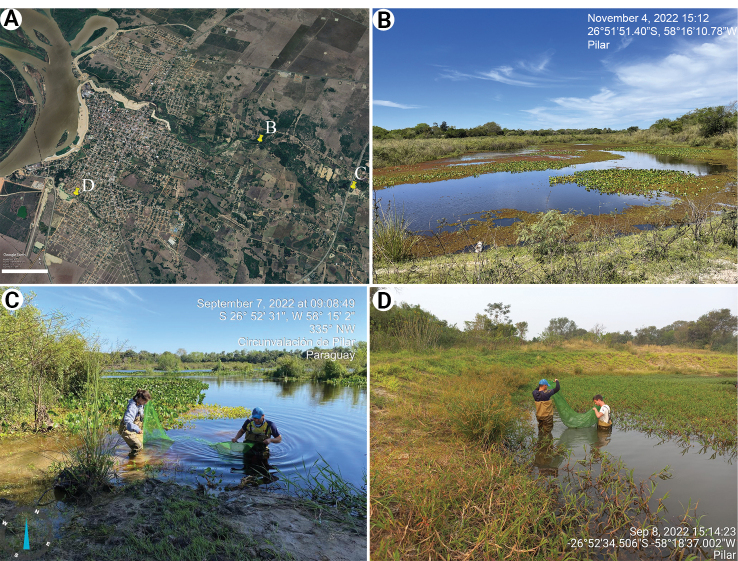
Localities of occurrence for *Hyalellamboitui* sp. nov. and *Hyalellajulia* sp. nov. in Pilar, southwestern Paraguay **A** map showing the position of the type localities **B–D** with scale: 900 m **B** Yegros Paso **C** Ring Road **D** San Lorenzo.

The geographical vicinity of the two new species and their distinct mandibular morphologies suggest that their differences may stem at least in part from trophic partitioning (Limberger et al. 2021). Distinct feeding habits may be tied to the different environments characterising the type localities of the two species (Fig. 13). Yegros Paso, the type locality of *H.julia* (Fig. 13B), falls within a complex of seasonal ponds with relatively stagnant waters bordering on a stream. Locally, water bodies expand and contract in cyclic dry and wet phases depending on rainfall levels (Hordijk et al. 2023). In contrast, the bodies of water in the type localities of *H.mboitui* (Ring Road and San Lorenzo; Fig. 13C, D) are characterised by somewhat stronger riverine influence, with more active flow regimes, and higher availability of macrophytes near the banks. Some distinctions in the morphology of their pleopod setae may suggest corresponding differences in locomotion. *Hyalellajulia* has denser, more strongly developed plumose setae on the pleopods that suggest a higher natatory capacity than in *H.mboitui*, and may make *H.julia* better adapted to swimming in lentic habitats (Streck et al. 2017). In contrast, *H.mboitui* may predominantly inhabit substrates in its lotic environment or remain near the river bank macrophytes.

The type locality of *H.julia* is managed for ongoing conservation and research projects on the endangered Pilar tuco-tuco (*Ctenomyspilarensis*). In contrast, major developments are scheduled or currently taking place at the type localities of *H.mboitui*, San Lorenzo and Ring Road, for the planned construction of flood defences. The connections between the bodies of water inhabited by *H.mboitui* and *H.julia*, and their seasonal continuity with the Ñeembucú River, preliminarily suggest that their area extends beyond the type localities. However, *Hyalella* is known for its high degree of endemism across South America, and the geographic range of different species in the genus is highly variable (Grosso and Peralta 1999; Streck et al. 2017). To map the ranges of *H.mboitui* and *H.julia*, and the degree to which ongoing developments may threaten the species survival, we recommend a wider sampling of freshwater invertebrates in the wetland complex around Pilar and more broadly in the Ñeembucú region.

The discovery of two new crustacean species, collected in a non-targeted impact assessment survey near an urban area, highlights the untapped potential of the Ñeembucú wetlands for biodiversity and conservation research. This ecologically important patchwork of rivers, streams, and flooded grasslands is severely understudied, and its invertebrate fauna remains virtually unexplored amid escalating anthropic impacts (Dickens et al. 2020; Mereles et al. 2020). More broadly, despite still comprising unfragmented areas of natural habitat, the Humid Chaco ecoregion in Paraguay is under increasing pressure from land use changes, resulting in high and rapid ongoing biodiversity losses (Mereles et al. 2020). Therefore, taxonomic studies are urgently needed to address the large remaining gaps in the scientific understanding of the region’s biodiversity. Our findings of two undescribed species provide supporting evidence of the potential presence of a significant number of undocumented taxa in the Ñeembucú wetlands, which are likely to benefit from habitat protection measures.

## Supplementary Material

XML Treatment for Hyalella mboitui

XML Treatment for Hyalella julia

## ﻿Additional information

### Author ORCIDs

Giovanni Mussini https://orcid.org/0009-0002-1093-5865

Gersey Vargas https://orcid.org/0000-0002-5625-8284

### Data availability

All of the data that support the findings of this study are available in the main text.
